# Genomic Surveillance of SARS-CoV-2 in Ibadan, Oyo State, Nigeria

**DOI:** 10.1093/cid/ciaf056

**Published:** 2025-07-22

**Authors:** Elizabeth T Akande, Adeola A Fowotade, Erkison Ewomazino Odih, Anderson O Oaikhena, Olasunkanmi Olisa, Gabriel Temitope Sunmonu, Boluwatife Adebiyi, Olabisi C Akinlabi, Oluwafemi A Popoola, Temitope O Alonge, Iruka N Okeke

**Affiliations:** Department of Pharmaceutical Microbiology, Faculty of Pharmacy, University of Ibadan, Ibadan, Nigeria; Biorepository Clinical Virology Laboratory, College of Medicine, University of Ibadan, Ibadan, Nigeria; University College Hospital and College of Medicine, University of Ibadan, Ibadan, Nigeria; Department of Pharmaceutical Microbiology, Faculty of Pharmacy, University of Ibadan, Ibadan, Nigeria; Department of Pharmaceutical Microbiology, Faculty of Pharmacy, University of Ibadan, Ibadan, Nigeria; Biorepository Clinical Virology Laboratory, College of Medicine, University of Ibadan, Ibadan, Nigeria; Department of Pharmaceutical Microbiology, Faculty of Pharmacy, University of Ibadan, Ibadan, Nigeria; Department of Pharmaceutical Microbiology, Faculty of Pharmacy, University of Ibadan, Ibadan, Nigeria; Department of Pharmaceutical Microbiology, Faculty of Pharmacy, University of Ibadan, Ibadan, Nigeria; University College Hospital and College of Medicine, University of Ibadan, Ibadan, Nigeria; Department of Community Medicine, College of Medicine, University of Ibadan, Ibadan, Nigeria; University College Hospital and College of Medicine, University of Ibadan, Ibadan, Nigeria; Department of Pharmaceutical Microbiology, Faculty of Pharmacy, University of Ibadan, Ibadan, Nigeria

**Keywords:** COVID-19, SARS-CoV-2, healthcare worker, Delta variant, Omicron variant, Nigeria, Ibadan

## Abstract

**Background:**

Oyo State, Nigeria, reported its first coronavirus disease 2019 (COVID-19) case on 21 March 2020 and subsequently recorded the fifth highest number of cases in Nigeria, most in the capital city Ibadan. We aimed to identify severe acute respiratory syndrome coronavirus 2 (SARS-CoV-2) variants that were circulating in Ibadan between August 2021 and August 2022 and to assess the utility of healthcare worker (HCW)–associated infections for endemic COVID-19 surveillance.

**Methods:**

Following ethical approval, SARS-CoV-2 real-time quantitative polymerase chain reaction (RT-qPCR)–confirmed samples were reverse-transcribed and sequenced on an Illumina MiSeq using ARTIC SARS-CoV-2 RT-PCR and sequencing protocols. Genomes were assembled using the nf-core/viralrecon pipeline. Quality control, phylogenetic, analysis, and variant identification were performed using publicly available software implemented in a custom Nextflow pipeline. Biodata and relevant clinical information were obtained from electronic case investigation forms.

**Results:**

We analyzed 258 samples with minimum non-N coverage of 70% and identified 12 SARS-CoV-2 lineages and 7 clades, all but one aligning with global lineages. Lineages BA.1 (22%) and BA.1.1 (48%) were the most common. Delta lineage predominated from August to September 2021, and was replaced by Omicron lineage from December 2021. Samples from HCWs (n = 60; 23%) accounted for a third (4 of 12) of all major lineages observed.

**Conclusions:**

We contributed 258 genomes from Oyo State to the GSAID repository and identified Delta and Omicron lineages circulating in Ibadan, in temporal alignment with global circulating lineages. As clinical testing declines HCWs are useful sentinels for genomic epidemiology capturing much of the diversity.

## Teaser

SARS-CoV-2 from COVID-19 patients in Ibadan were predominantly Delta variants (Aug-Sept 2021), and Omicron variants (Dec 2021-July 2022). The commonest lineages were recovered from health workers, who could be employed for variant tracking now that extensive testing has ceased.

Severe acute respiratory syndrome coronavirus 2 (SARS-CoV-2) is the causative agent of the coronavirus disease 2019 (COVID-19) pandemic. COVID-19 primarily manifests as an upper respiratory tract infection, and its definitive diagnosis relies on real-time polymerase chain reaction (RT-PCR) assays for SARS-CoV-2 nucleic acid detection in respiratory specimens [1]. Various factors, including age, underlying health conditions, and immunological status, influence the clinical course and severity of COVID-19.

The predominant mode of SARS-CoV-2 transmission is through respiratory droplets, with the potential for airborne transmission in specific situations, such as in confined spaces and during medical procedures that generate aerosols [2, 3]. Additionally, transmission through fomites has been reported [4]. On 30 January 2020, the World Health Organization (WHO) declared COVID-19 a public health emergency of international concern. Subsequently, on 11 February 2020, it was formally recognized as a new coronavirus disease, and on 11 March 2020, the WHO declared it a pandemic [5].

As of October 2023, Nigeria had reported nearly 270 000 confirmed COVID-19 cases and 3155 deaths, with 10 352 confirmed cases in Oyo State. Nigeria's COVID-19 cases have largely been identified in Lagos and Abuja, with local transmission at these locations seeded from importations by travelers [6]. These commercial and administrative capitals as well as 5 of Nigeria's other 36 states account for 75% of the national COVID-19 case load. Oyo State is one of them, and its residents have been exposed early and often to new SARS-CoV-2 introductions. Oyo State recorded 202 of Nigeria's 3155 deaths formally attributed to COVID-19.

Healthcare workers (HCWs) are at an elevated risk of exposure to the virus due to their proximity to infected patients. According to data published by the WHO, 322 vaccine candidates were in development as of 22 October 2021 [7]. As of 13 June 2022, approximately 122 COVID-19 vaccines were undergoing human clinical trials, trials were almost finished for 49 vaccines, and 12 vaccines had been authorized for use in humans [8]. The uptake of these vaccines has been subpar globally [9] despite the approval of some of these COVID-19 vaccines for the prevention of the COVID-19 disease (the BNT162b vaccine produced by Pfizer and the ChAdOx1 nCoV-19 vaccine produced by Oxford–AstraZeneca, Moderna, Sputnik V, and Johnson & Johnson). During the early stages of the COVID-19 pandemic, vaccine availability in Nigeria was limited due to the country's large population. Through the Covid-19 Vaccines Global Access (COVAX) initiative, Nigeria received its first allocation of the AstraZeneca vaccine on 2 March 2021, with HCWs prioritized in the initial rollout [10]. In Oyo State, the vaccination campaign officially commenced on 24 March 2021 in Ibadan, marking the start of the state's efforts to immunize its population against COVID-19 [10]. Within Oyo State, HCW caseload and death toll due to COVID-19 were comparatively high, and the health-facility focus on the disease reduced access to other types of routine care [11].

During the course of the pandemic, SARS-CoV-2 evolved a number of genetic variants, some of which are of concern because they evade vaccines or diagnostics or because of enhanced virulence. Understanding SARS-CoV-2 variant epidemiology in Oyo State is important for determining the transmission trajectories that lead to, and arise from, endemic COVID-19. We therefore undertook SARS-CoV-2 variant identification of a subset of positive cases using Illumina sequencing. Additionally, now that community testing has declined, we sought to determine whether infected HCWs could serve as sentinels to detect new genetic variants and implement effective measures to mitigate the virus's spread.

Our aim in this study was to enhance virus surveillance by analyzing SARS-CoV-2 genomes, comparing local and global variants, evaluating HCWs as representatives of the population, and investigating genetic variations in Oyo State, Nigeria.

## METHODS

### Sample Collection and Processing

This study was conducted in Ibadan, the capital city of Oyo State, located at latitude 7.402°N and longitude 3.917°E. Ibadan houses Nigeria's first teaching hospital, one of the biggest tertiary referral centers in the southwest region of Nigeria and the entire country. PCR-confirmed COVID-19–positive samples collected from Ibadan testing centers and analyzed at the Biorepository Clinical Virology Laboratory (BCVL), College of Medicine, University of Ibadan, from August 2021 through December 2022 were sequenced. BCVL is a biosafety level 2 molecular diagnostic laboratory accredited by the Nigerian Centre for Disease Control (NCDC) for COVID-19 testing.

Demographic data, including age, sex, occupation, and name of vaccine taken, if any, were retrieved from the electronic central database, NCDC website. Nasopharyngeal and oropharyngeal swabs collected from all local government areas in Oyo State were analyzed for SARS-CoV-2 using RT-PCR at BCVL. All samples were handled with appropriate biosafety measures from the point of collection and transport to laboratory analysis. Samples were placed into a reaction buffer and inactivated by addition of absolute ethanol. Nucleic acid was extracted using the QIAamp Viral RNA kit (QIAGEN, Germany) according to manufacturer's protocol [12]. Extracted viral nucleic acid was subsequently reverse-transcribed and screened for the presence of SARS-CoV-2 using the ARTIC SARS-CoV-2 RT-PCR Module, E7626L (NEBNext). An ambidirectional (retrospective and prospective) approach was used, and selected positive samples underwent library preparation using the Ultra II FS DNA library prep kit for Illumina (NEBNext, E7805L) following the ARTIC protocol, which is available online [13], and were optimized in the laboratory before Illumina sequencing was conducted.

### Bioinformatics Analysis

Quality control and sequence assembly were carried out using the viralrecon analysis pipeline, which is freely available online (https://github.com/nf-core/viralrecon) [14]. Downstream analysis of the assembled sequences, including quality filtering, lineage, and clade assignment with Pangolin v3.1.20 [15] and Nextclade (v1.10.3 [16]), and phylogenetic tree construction with IQ-TREE (v2.0.3 [17]) were performed using an adapted, custom-built pipeline, also available online (https://gitlab.com/bioinfo_erkison/sarscov2). The generated phylogenetic tree was annotated and visualized in Microreact (https://microreact.org [18]) and is available online https://microreact.org/project/vAmqHRVYsnKMnZsL9p4Ubf-updatedgeneral. Assembled sequences that passed quality checks were deposited in the Global Initiative on Sharing All Influenza Data (https://gisaid.org/ [19]).

### Ethical Considerations

Ethical approval for the study was obtained from the joint University of Ibadan/University College Hospital Ethics Committee before commencement of the study. Informed consent was waived as the patients were not dealt with directly, and confidentiality was maintained by the removal of participant identifiers from the dataset.

## RESULTS

**Figure 1.**
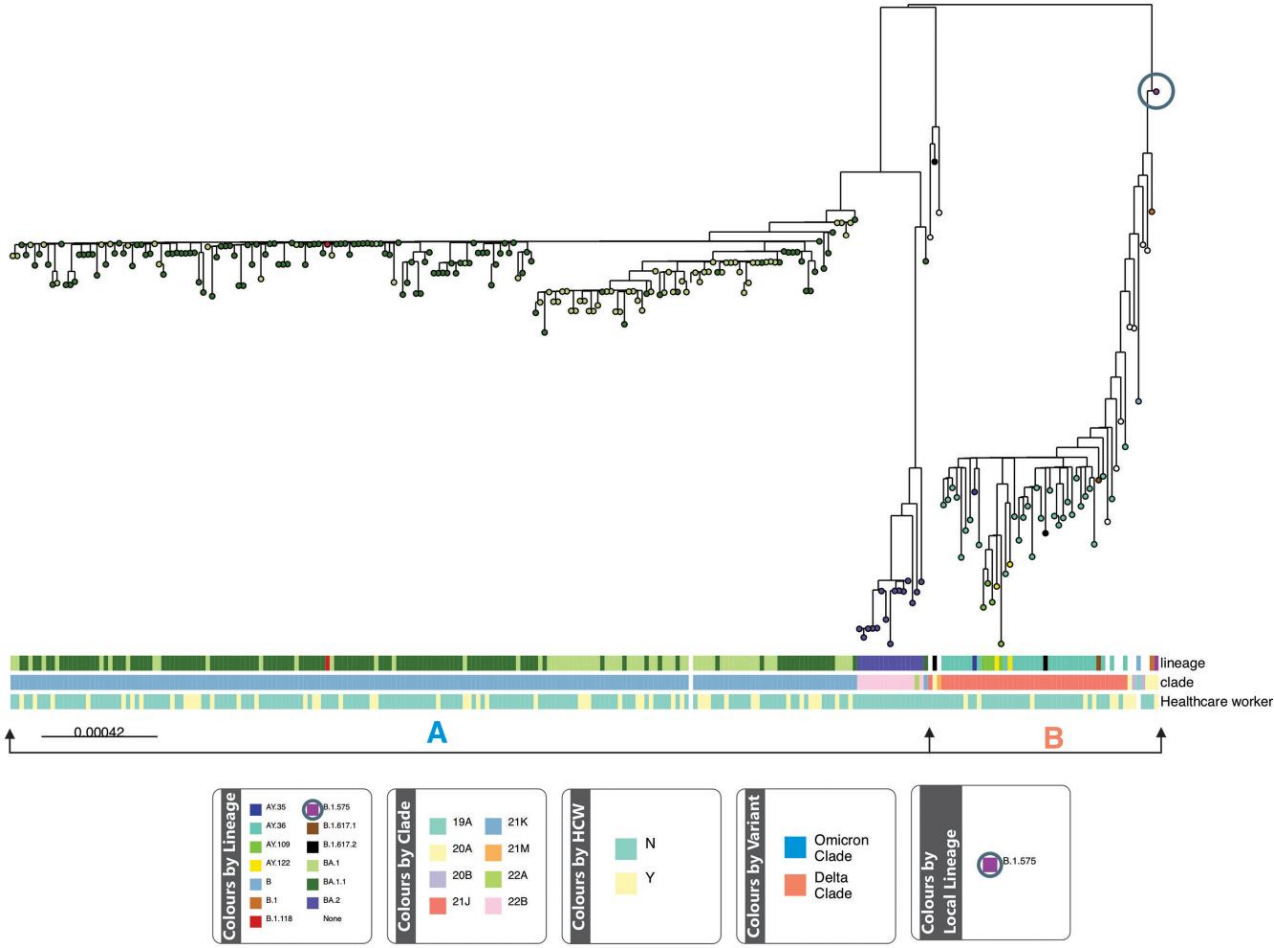
The 2 major clades found during the study period: clade A (Omicron variants, including BA.1, BA.1.1, BA.2 lineages) and clade B (Delta variant lineages). The leaf at the top left consists primarily of unclassified lineages found during the study. The circled lineage (B.1.575) at the top right had unique mutations and was rare.

A total of 258 samples collected between August 2021 and September 2022 from 13 local government areas in Ibadan, Oyo State, were analyzed. The samples included 2 samples from Osun State; 4 from Oyo Town, Oyo State; 1 from Niger State; and 1 from Lagos State; all were retrieved from patients who visited Oyo State specimen collection centers. There were 2 major clades, 1 smaller clade that harbored the Delta variants and a larger clade that harbored the Omicron variants with a total of 12 lineages. The smaller clade (based on number of constituent clades), denoted as clade A, comprised 7 lineages (B.1.118, BA.1.1, B.1.575, B.1.617.2, B.1, BA.1, and BA.2), while the other clade, denoted as clade B, comprised 5 lineages (AY.36, AY.35, AY.122, AY.109, and B.1.617.2; Figure 1). We also observed 9 nonassigned lineages clustered within the Delta clade.

**Figure 2.**
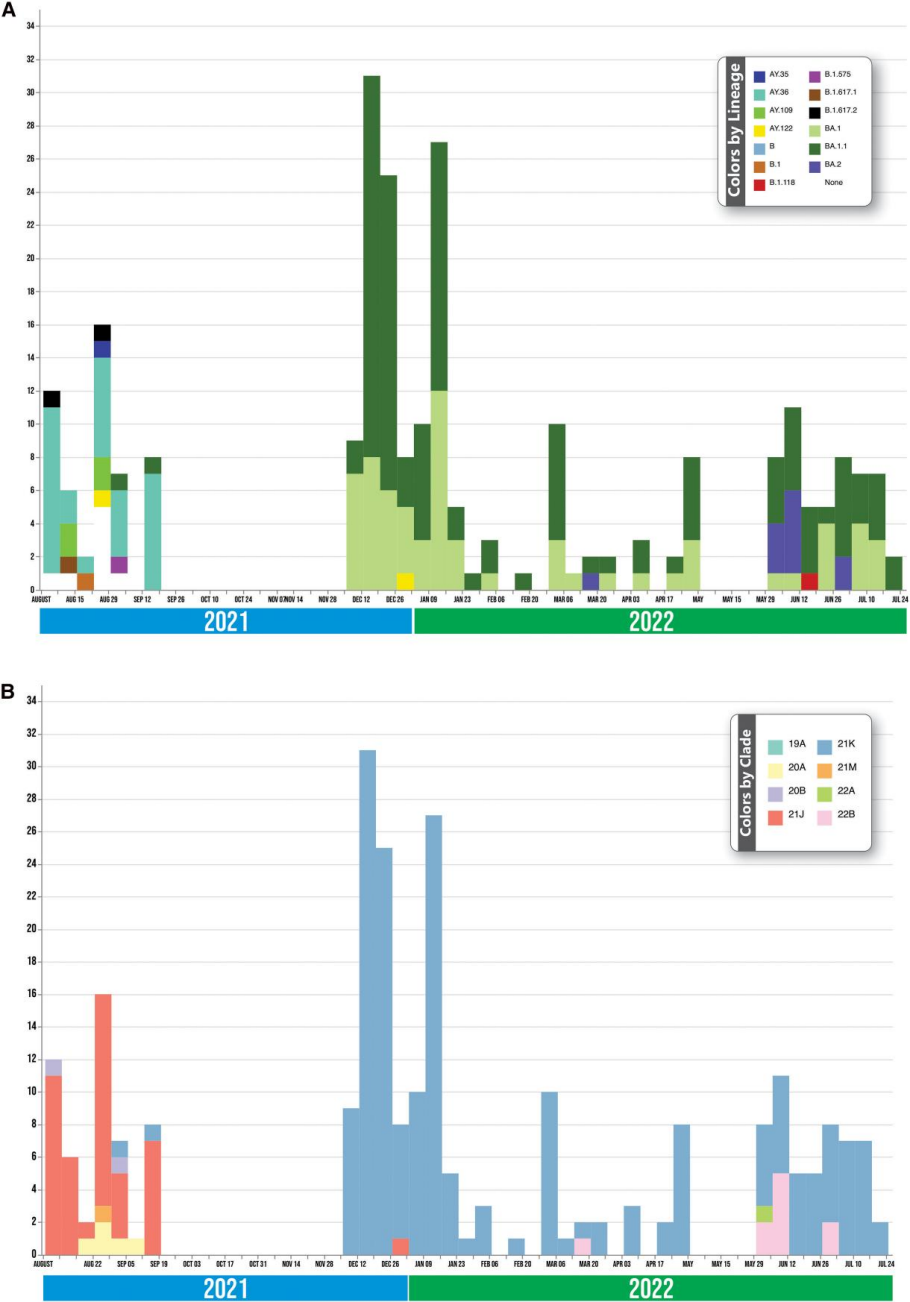
Epicurve showing the severe acute respiratory syndrome coronavirus 2 clades identified during the study period. Twelve lineages were identified during the study period (*A*). Delta AY.36 was the most predominant lineage at the start of the surveillance period and was displaced in a new wave by Omicron BA.1 and BA.1. (*B).* Distribution of the various lineages in the clades. The highest clade, 21K, harbored the Omicron lineages, and 21J harbored most of the Delta lineages.

Clades 21J (AY.36, AY.35, AY.122, AY.109, B.1.617.2, B.1.617.1), 20A (B.1.575, B.1.617.2, B.1), 21M, and 20B circulated concurrently before they became displaced in December by clades 21K (BA.1, BA.1.1, B.1.118), 22B (BA.2), and 22A (BA.2; Figure 2). AY.36 was the most common Delta variant detected in this study.

**Figure 3.**
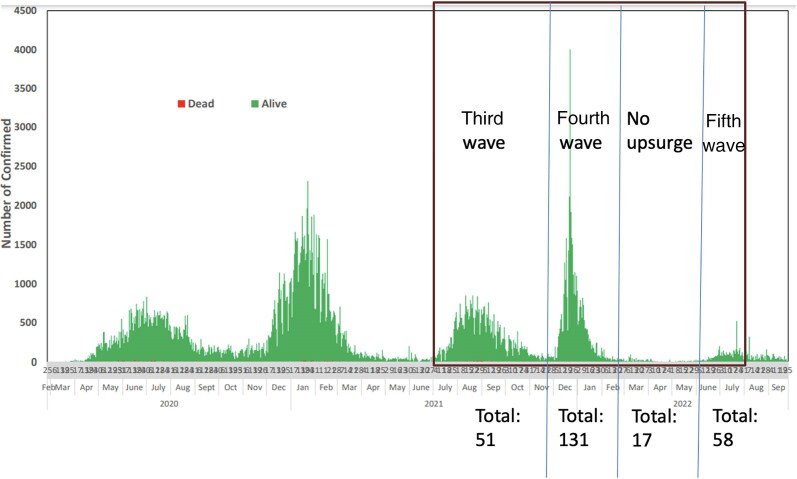
Epicurve of the waves in Nigeria and sample retrieval periods. Samples were retrieved between the third and fifth waves experienced in Nigeria. Included are the numbers per lineage retrieved per wave in Oyo State.

Samples sequenced in this study were collected during the third through fifth waves (between August 2021 and December 2022) of COVID-19 experienced globally (Figure 3). Most of the lineages that were identified coincided with those that were disseminating globally at the time. However, the study uncovered a retrospective B.1.575 genome in September 2021 from a sick HCW with a history of prior complete vaccination with the Oxford–AstraZeneca vaccine.

**Figure 4.**
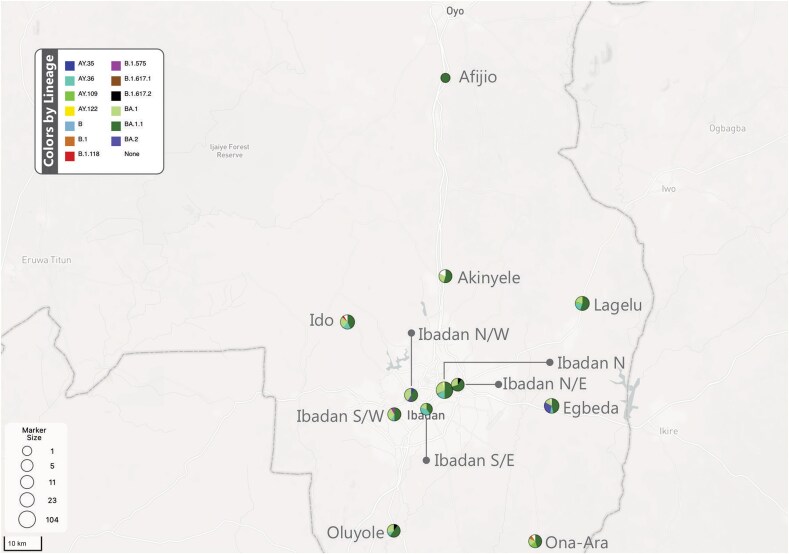
Distribution of lineages across the 12 local government areas in Oyo State covered in this study. BA.1.1 was present in all local government areas; the highest was recorded from Ibadan North. Abbreviations: IBN, Ibadan-North; IBNE, Ibadan North-East; IBNW, Ibadan North-West; IBSE, Ibadan South-East; IBSW, Ibadan South-West.

BA.1.1 was prevalent across all 12 local government areas within Ibadan, accounting for 49% of circulating lineages. BA.1 was detected in 11 of the 12 local government areas and represented 25% of all circulating lineages. Notably, AY.36 emerged as the predominant Delta lineage variant, dominating in 7 of the 12 local government areas (12% of circulating lineages) within the study population and timeframe (Figure 4).

### Lineages Detected in the HCW Subpopulation

**Figure 5.**
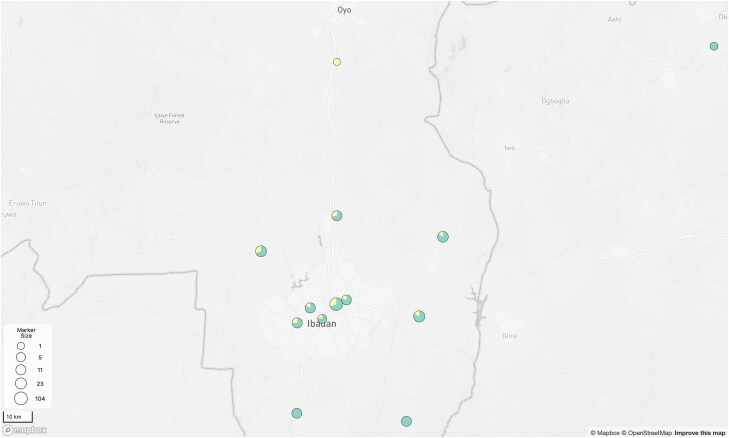
Geographical distribution of healthcare workers (HCWs) by local government area. The proportion of samples that came from HCWs in each local government area is indicated in yellow. Three of the 12 local government areas within Ibadan had no representative HCWs and are marked by compleletely green circles.

**Figure 6.**
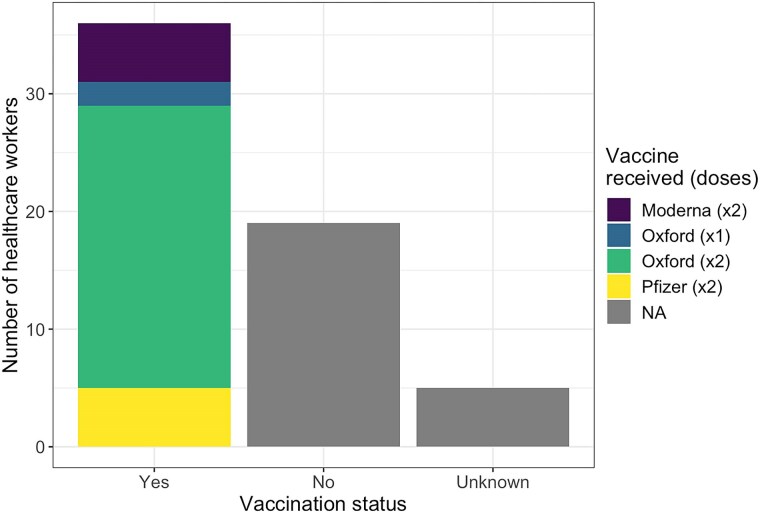
Vaccination status and vaccine type for tested healthcare workers (HCWs). Most HCWs were fully vaccinated with the Oxford–AstraZeneca vaccine. Abbreviation: NA, not applicable.

Sixty of the samples taken were obtained from HCWs, accounting for 23% of the entire sampling population. Of the 12 local government areas represented, 10 had at least 1 sample from an HCW (Figure 5). At least 1 HCW sample was found within 4 of the 7 clades identified, including 20A (n = 2 of 5), 20B (n = 1 of 2), 21J (n = 8 of 43), and 21K (n = 49 of 192). The remaining 3 clades (1 with 14 genomes and 2 with 1 genome each) had no HCW representative. Clade 21K had the highest percentage (82%) of HCW samples (Table 1). Thirty-six (60%) of the HCWs were vaccinated, 94% (n = 34) of whom were fully vaccinated. Of the 34 fully vaccinated HCWs, 24 received the Oxford–AstraZeneca vaccine, 5 received the Pfizer vaccine, and 5 received the Moderna vaccine. The remaining 2 vaccinated HCWs received a single dose of the Oxford–AstraZeneca vaccine. Nineteen HCWs (32%) were not vaccinated, and the vaccination status of 5 HCWs (8%) was unknown (Figure 6).

**Table 1.** Distribution of Healthcare Worker Specimens Among Clades and Lineages Identified in This Study

Clade Division	Clade	Lineage	Number of Genomes	Number (%) of Genomes From HCWs	% of HCW Genomes in Clade
Clade A	21M	None	1	0 (0)	0
	22A	BA.2	1	0 (0)	0
	22B	BA.2	14	0 (0)	0
	21K	BA.1, BA.1.1, B.1.118	192	49 (26)	82
	20A	B.1, B.1.575, B.1.617.2, none	5	2 (40)	3
Clade B	20B	BA.2, none	2	1 (50)	2
	21J	AY.36, AY.35, AY.122, AY.109. B.1.617.2, none	43	8 (19)	13
All clades	…	…	258	60 (23)	100

Abbreviation: HCW, healthcare worker.

## DISCUSSION

An earlier study [20] found that the Alpha “variant of concern” and the Eta lineage predominated between July 2020 and August 2021 in Ibadan. In this study, which covered the third (July 2021–November 2021), fourth (December 2021–January 2022), and fifth COVID-19 waves (June 2022–August 2022) in Nigeria and spanned the October 2021 global dissemination of Omicron [21], the SARS-CoV-2 Delta and Omicron variants were detected in Ibadan, Oyo State.

The Delta variant with lineages AY.36, AY.35, AY.122, AY.109, and B.1.617.2 spanned the period between August 2021 and September 2021 during the third wave of the pandemic but was quickly replaced by the Omicron variant in the fourth wave with lineages BA.1, BA.1.1, and BA.2 from December 2021 to July 2022 as in Ilorin, North Central Nigeria [22], and also across Africa where Delta variants were predominant in the third wave but displaced by Omicron variants in the fourth wave [23]. The Omicron variant has an effective reproduction number and basic reproduction number of 3.8 and a transmissibility that was 2.5 times higher than that of the Delta variant [24]. This likely accounts for the swift surge in Omicron cases, although Omicron's augmented immune evasion, facilitated by multiple mutations in the Spike protein, may also have contributed. A comparative study of household contacts of Delta- and Omicron-infected patients revealed a higher secondary attack rate with Omicron, especially among fully vaccinated and boosted contacts [25]. The data show that major globally disseminated clades were rapidly disseminated to and through Ibadan. The AY.36 lineage was the most predominant during the third wave, which is in agreement with a previous report by Ozer et al [20] that stopped sampling the same month this study's retrospective sampling commenced. BA.1.1, a sublineage of BA.1, was the predominant Omicron variant during the fourth wave and was present in all 12 local government areas in this study. This is not surprising because this lineage possessed additional mutations over BA.1 that increased its transmissibility [26].

Our study expands on the study by Ozer et al [20] who examined SARS-CoV-2 variants circulating in Ibadan between July 2020 and August 2021. While only a fraction of viruses from the 270 000 confirmed cases of COVID-19 in Oyo State were sequenced in both studies (a total of 636), they demonstrated that major SARS-CoV-2 lineages and variants of interest, including Wuhan, Alpha, Beta, Gamma, B.1.525 (Eta), and B.1.1.318 [27], which were not detected in this study, had been completely replaced by Delta variants [20]. Our own work shows that Omicron lineages then completely displaced Delta variants. Most of the lineages detected in the current study were found globally, but the B.1.575 variant largely circulated within the Pamplona region of Spain [28] and thus was not expected to be present in Ibadan. Notably, existing literature offers no reports of B.1.575 in Nigeria and limited global occurrence. The patient from whom this variant was recovered had no history of international travel but affirmed local travel to Abeokuta in Ogun State, Nigeria, which, like Ibadan, is proximal to Lagos but has no direct international connection. Consequently, the reasonable inference is that at least 1 instance of domestic transmission contributed to our detection of that lineage. The B.1.575 variant was detected in an HCW.

COVID-19 testing in Nigeria declined from the beginning of this study and remains at very low levels today. We therefore used an ambidirectional approach to identify variants in both prospective and retrospective samples, with the objective of determining whether HCWs could serve as sentinels for variant emergence in Ibadan. Viruses from HCWs made up 23% of those sequenced. We found 7 clades, 3 of which had no HCW sample representative. In addition to the small number of patients sampled in Oluyole and Ona Ara local government areas, this likely arose from the fact that these clades predominated in local government areas with fewer hospitals [29]. In Ibadan North, Ibadan Northeast, and Ibadan Southwest, which contain 2 or more large general or teaching hospitals, a large number of cases among HCWs were recorded.

The small number of cases among HCWs can also be traced to protection from vaccines, which were predominantly available to HCWs before this study started. Additionally, there is a noteworthy 80% rate of vaccine administration and collection among HCWs in Oyo State [30].

## CONCLUSIONS

The results from this study suggest that the circulating variants in Ibadan largely mirror circulating variants globally. However, the unexpected detection of B1.575, without informative context, suggests the necessity for more rigorous surveillance efforts to unveil potential low-level lineage circulation that had hitherto been deemed absent within the country's boundaries. Furthermore, this observation underscores the imperative of promptly identifying and instituting epidemiological containment strategies for any emerging variants that may possess a heightened potential for successful propagation in the future. This calls for heightened vigilance and monitoring of the evolving landscape of viral variants to safeguard public health.

## Notes

***Acknowledgments.*** We thank members of the Oyo State Emergency Operations Centre who coordinated sample collection and transport to the Biorepository Clinical Virology Laboratory. We are grateful to COVID-19 Genomics UK Consortium (COG-UK)-Train and Wellcome connecting science for training opportunities that made it possible for our bacteriology laboratory to engage in severe acute respiratory syndrome coronavirus 2 sequencing and to Anthony Underwood, Varun Shamanna, and David M. Aanensen for helpful comments.

***Disclaimer.*** The views expressed here are those of the authors and not necessarily those of the funders.

***Financial support***. This work was supported by the Gates Foundation (INV-036519) and leveraged instrumentation from Official Development Assistance funding from the National Institute of Health Research (16/136/111) for the Global Health Research Unit for Genomic Surveillance of Antimicrobial Resistance. I. N. O. is a Calestous Juma Science Leadership Fellow supported by the Gates Foundation (INV-036234).

***Supplement sponsorship.*** This article appears as part of the supplement “COVID-19 Vaccine Research in Africa,” sponsored by the International Vaccine Institute and funded by the Coalition for Epidemic Preparedness Innovations (CEPI).
